# Supplementary material for: The bacterial quorum sensing signal DSF hijacks Arabidopsis thaliana sterol biosynthesis to suppress plant innate immunity
Source: Life Sci Alliance. 2020 Aug 11;3(10):e202000720. doi: 10.26508/lsa.202000720 (PMC7425213; doi:10.26508/lsa.202000720)
Supplement: Supplementary file 3 [file LSA-2020-00720_TableS1.docx]

**Table S1. Key resources table**

| **REAGENT or RESOURCE** | **SOURCE** | **IDENTIFIER** |
| --- | --- | --- |
| **Antibodies** | | |
| Rabbit Polyclonal anti-GFP | Torrey Pines BioLabs | Cat#TP401 |
| Rabbit Monoclonal anti-Phospho-p44/42-MAPK | Cell Signalling Technology | Cat#4370S |
| Mouse anti-FLAG M2 antibody | Sigma | Cat#A8592 |
| Rabbit anti-HA (3F10) antibody | Roche | Cat#12013819001 |
| Mouse anti-FLAG M2 affinity gel | Sigma | Cat#A2220 |
| **Bacterial and Virus Strains** | | |
| *Pseudomonas syringae* pv. *tomato* (*Pst*) DC3000 | 1 | N/A |
| **Chemicals, Peptides, and Recombinant Proteins** | | |
| *cis*-11-methyl-dodecenoic acid (DSF) | Sigma-Aldrich | Cat#42052 |
| ES9-17 | Chembridge | Cat#7577817 |
| Brefeldin A (BFA) | MPBio | Cat#159027 |
| Wortmannin | Sigma-Aldrich | Cat#W1628 |
| Lovastatin | Santa Cruz Biotech | Cat#SC-200850 |
| Methyl-*β*-cyclodextrin (M*β*CD) | MedChemExpress | Cat#HY-101461 |
| *β−*Sitosterol | Abcam | Cat#ab143122 |
| Stigmasterol | Santa Cruz Biotech | Cat#SC-281156 |
| Campesterol | Santa Cruz Biotech | Cat#SC-214658 |
| flg22 (QRLSTGSRINSAKDDAAGLQIA) | GL Biochem Ltd. | N/A |
| flgII-28 (ESTNILQRMRELAVQSRNDSNSATDREA) | GL Biochem Ltd. | N/A |
| elf26 (ac~SKEKFERTKPHVNVGTIGHVDHGKTT) | GL Biochem Ltd. | N/A |
| **Critical Commercial Assays** | | |
| Rneasy Plant Minikit | Qiagen | Cat#74904 |
| RNAClean XP Kit | Agencourt | Cat#A63987 |
| SuperScript III First-strand synthesis system | Invitrogen | Cat#18080051 |
| Kapa SYBR FAST qPCR Master Mix (2X) Universal | Kapa Biosystems | Cat#KK4601 |
| Spurr Low-viscosity embedding kit | Sigma-Aldrich | Cat#EM0300 |
| **Experimental Models: Cell Lines** | | |
| Hamster: CHO cells: GFP-GPI | 2 | N/A |
|  |  |  |
| **Experimental Models: Organisms/Strains** | | |
| *Arabidopsis:* Col-0 | ABRC | CS1092 |
| *Arabidopsis:* RabF2b-YFP (Wave_2Y) | ABRC | CS781647 |
| *Arabidopsis:* Got1p homolog-YFP (Wave_18Y) | ABRC | CS781655 |
| *Arabidopsis*: FLS2-GFP | 3 | N/A |
| *Arabidopsis*: *pREM1.2::YFP:REM1.2* | 4 | N/A |
| *Arabidopsis*: Ler | 5 | N/A |
| *Arabidopsis*: *fkX-224* | 5 | N/A |
| *Arabidopsis*: *Ws* | 6 | N/A |
| *Arabidopsis*: *smt1-1* | 6 | N/A |
| *Arabidopsis*: BOR1-GFP | 7 | N/A |
| *Arabidopsis:* BRI1-GFP | 8 | N/A |
| *Arabidopsis:* CLC-GFP | 9 | N/A |
| **Oligonucleotides** | | |
| *EF1α* F: TGAGCACGCTCTTCTTGCTTTCA | 10 | N/A |
| *EF1α* R: GGTGGTGGCATCCATCTTGTTACA | 10 | N/A |
| *FRK1F:* ATCTTCGCTTGGAGCTTCTC | 11 | N/A |
| *FRK1R:* TGCAGCGCAAGGACTAGAG | 11 | N/A |
| ***Plasmids*** |  |  |
| pHBT-FLS2-HA | 12 | N/A |
| pHBT-BAK1-FLAG | 12 | N/A |
| **Software and Algorithms** | | |
| Matlab | MathWorks | RRIS:SCR_001622 |
| Imaris | Bitplane | RRID:SCR_007370 |
| Fiji (ImageJ) | NIH | RRID:SCR_002285 |
| Graphpad PRISM | Graphpad | Ver. 7.0, RRID:SCR_002798 |
| Huygens | Scientific Volume Imaging | RRID:SCR_014237 |
| **Other** | | |
| Tecan plate reader | TECAN LifeSciences | Infinite M200Pro |
| AB StepOnePlus Real-Time PCR system | Applied Biosystem | Cat#4376600 |

**References**

1. D. A. Cuppels, Generation and characterization of Tn5 insertion mutations in *Pseudomonas syringae* pv. *tomato*. *Appl. Environ. Microbiol.* **51**, 323-327 (1986).

2. R. Raghupathy *et al.*, Transbilayer lipid interactions mediate nanoclustering of lipid-anchored proteins. *Cell* **161**, 581-594 (2015).

3. S. Robatzek, D. Chinchilla, T. Boller, Ligand-induced endocytosis of the pattern recognition receptor FLS2 in *Arabidopsis*. *Genes & development* **20**, 537-542 (2006).

4. I. K. Jarsch *et al.*, Plasma membranes are subcompartmentalized into a plethora of coexisting and diverse microdomains in Arabidopsis and Nicotiana benthamiana. *The Plant Cell* **26**, 1698-1711 (2014).

5. K. Schrick *et al.*, FACKEL is a sterol C-14 reductase required for organized cell division and expansion in Arabidopsis embryogenesis. *Genes & Development* **14**, 1471-1484 (2000).

6. A. C. Diener *et al.*, Sterol methyltransferase 1 controls the level of cholesterol in plants. *The Plant Cell* **12**, 853-870 (2000).

7. J. Shen *et al.*, A plant Bro1 domain protein BRAF regulates multivesicular body biogenesis and membrane protein homeostasis. *Nature communications* **9**, 3784 (2018).

8. N. Geldner, D. L. Hyman, X. Wang, K. Schumacher, J. Chory, Endosomal signaling of plant steroid receptor kinase BRI1. *Genes & development* **21**, 1598-1602 (2007).

9. L. Fan *et al.*, Dynamic analysis of Arabidopsis AP2 σ subunit reveals a key role in clathrin-mediated endocytosis and plant development. *Development* **140**, 3826-3837 (2013).

10. T. Czechowski, M. Stitt, T. Altmann, M. K. Udvardi, W.-R. Scheible, Genome-wide identification and testing of superior reference genes for transcript normalization in *Arabidopsis*. *Plant Physiol.* **139**, 5-17 (2005).

11. P. He *et al.*, Specific bacterial suppressors of MAMP signaling upstream of MAPKKK in *Arabidopsis* innate immunity. *Cell* **125**, 563-575 (2006).

12. L. Xu, X. Yao, N. Zhang, B.-Q. Gong, J.-F. Li, Dynamic G protein alpha signaling in *Arabidopsis* innate immunity. *Biochem. Biophys. Res. Commun.* (2017).
